# Some Experiences in Surgery of the Appendix with Report of Cases

**Published:** 1909-03

**Authors:** R. R. Kime

**Affiliations:** Atlanta, Ga.; Gynecologist and Abdominal Surgeon to Tabernacle Infirmary


### SOME EXPERIENCES IN SURGERY OF THE APPENDIX WITH REPORT OF CASES.

BY R. R. KIME, M. D., ATLANTA, GA.

GYNECOLOGIST AND ABDOMINAL SURGEON TO TABERNACLE INFIRMARY.

There is nothing more uncertain than the findings in surgery of the appendix. Its position, structure, and relation to other organs of the abdominal and pelvic cavities as well as relation to bone and muscular structures account for the varied conditions and complications that arise in connection with the appendix.

We shall not endeavor in this paper to mention or illustrate all the varied conditions, complications and sequlae of appendicitis, but simply present a few of the interesting conditions we have lately encountered in our surgical work.

Case 1.—Tubular appendicitis with complete destruction of the appendix—Mrs. B., age 30 years, widow, husband died 4 or 5 years previous of pulmonary tuberculosis.

Patient has had several attacks of pain in right inguinal region—of late more or less constant pain, tenderness, indigestion and loss in weight with induration and enlargement in right side.

Operation at Tabernacle Infirmary, November, 1907. Incision at border rt. rectus muscle. Adhesions of omentum separated a portion 3 by 4 inches removed, adhesion of small intestine separated a thickened indurated point cleansed with hydrogen-dioxide—pure carbolic acid applied, then neutralized with alcohol followed with salt solution, the surface closed over with catgut sature.

At end of caecum appendix obliterated—a mass two by two and one-half inches the thickened degenerated wall of the intestine was curetted off. Hydrogen-Dioxide applied followed with carbolic acid pure neutralized with alcohol and salt solution. Surface closed over with catgut sutures, incision closed by layer sutures of catgut and Murphy fig. 8—black silk worm gut suture. Pathologist reported tuberculosis. Patient made uninterrupted recovery rapidly gained in health and flesh and has since remained in good health.

Case 2.—Atrophy or obliterating appendicitis—movable rt. kidney—nephritis, dysmenorrhoea, anteflexed uterus.

Miss B. from North Georgia, age 24 years, injured in a runaway accident six months previous, being thrown from buggy. Pain and tenderness of rt. kidney and in region of appendix and rt. ovary. Hyalinegran. casts, round ep. in urine, no alb. Operation July, 1908 at Tabernacle Infirmary. Incision one-half inch below 12th rib. Decapsulated and stitched up rt. kidney, dilated and curetted uterus, incision made transverse in angle of flexion and closed longitudinal with uterus, then abdomen was opened at border rt. rectus muscle. Rt. ovary resected and after considerable search appendix was found very little over one inch in length and one-third the normal diameter and very little meso-appendix. The appendix was removed and abdominal incision closed with catgut and fig. 8 silk worm gut sature. There wa ssome little infection from knots of chronic catgut in kidney incision otherwise patient made a good recovery. Her mother made special request not to remove appendix because five of the immediate family had died from appendicitis, two with and three without operation.

Case 3.—Chronic appendicitis, general tenderness of bowels and obstinate constipation, indigestion. With tonics and medicine to improve digestion, large doses of laxatives all tenderness would disappear to return as soon as bowels became constipated.

When worse gran. and hyaline casts, kidney epithelium and large amounts of indican were found in urine. Was living with sister who has a severe attack of pulmonary tuberculosis.

Patient, Mr. G., age 24 years, single. Operated, December, 1908 at Tabernacle Infirmary. Incision—muscle splitting method, omentum, caecum and appendix inflamed, concretion in tip of appendix retrocaecal, meso appendix short—in stitching and tying off, had free bleeding, extravisation of blood under peritoneal covering forming small hematoma controlled by lateral lapping and stitching, abdominal incision closed with layer cat gut satures. About one week after operation one or two glands became inflamed in left ingunial region but soon subsided.

Patient is gaining rapidly and has gone to the country to rest and recuperate. A late letter states that he is doing well and gaining flesh rapidly.

Case 4.—Long Retro—caecal appendix—no meso appendix—concretion in tip.

Miss G., age 23 years, subject to attacks of rheumatism, marked retroversion of uterus, dysmenarrhoea, confined to bed at times. Tenderness in right and left Morris points and McBurney point, right kidney movable 1st degree and tender granular epithetial and hyaline casts, rd. ep. and some indican in urine. Regulation of diet giving bitter tonics, intestinal and renal antiseptics with mild alkaline diuretics kidney function very much improved. Operation December, 1908 at Tabernacle Infirmary. Cervix dilated, uterus curetted, median incision in abdomen, both ovaries resected, both round ligaments with anterior layer of broad ligaments plicated anteriorly method of Coffee, appendix could not be brought up about 5 inches long retrocaecal without meso-appendix had to be separated from caecum commencing at tip in which was a concretion size of grape seed, stump treated in usual manner, appendix and caecum congested, abdominal incision closed with layer cat gut and figure 8 silk worm gut sature.

Patient made a good recovery and kidney lesion clearing up.

Case 5.—Appendicitis due to oxyuris vermicularis (thread-worm).

Patient, F. C., boy, age 10 years. Taken with pain in right inguinal region a. m. of May 15, 1908. Dr. Brawner saw case at 7:30 p. m.—Diagnosed appendicitis. I was called at 8:30 p. m.

Patient was transferred to Wesley Memorial Hospital and appendix removed before 10 p. m., by muscle splitting operation.

Appendix four or five times normal size, badly inflamed, ready to necrose, was occluded at caecal end and contained several thread-worms.

Aether pneumonia developed within 48 hours, which under appropriate treatment soon subsided. The coughing produced a little irritation of incision, otherwise patient made uneventful recovery.

Case 6.—Mrs. S., age 24 years, married, one child 10 months old—first attack of appendicitis—seven days duration was better, temperature and pulse nearly normal, grew worse in the afternoon. Dr. Vaughn, the attending physician was called and recognized a marked change for the worse. I was called, found patient with temperature 100 F., pulse 140, weak—no special pain, some rigidity muscles right side, bowels distended, some tenderness, anxious expression, advised immediate operation. Hypodermic of hyoscine and morphine given at 8:30 p. m., patient moved at once in elevated position to Tabernacle Infirmary.

Incision in right iliac region—abscess not ruptured, peritoneal cavity packed off with gauze—abscess opened and drained with rubber tubes, gauze wick in one, soiled pads removed and light gauze packing placed to protect peritoneal cavity from abscess drainage, appendix not found—operation and dressing consumed 15 minutes. Fecal fistula followed, surfaces sloughed from irritating discharge. Fistula closed, wound healed, no hernia, patient well and has regained usual weight.

Case 7.—Mr. B., age 46, weight 225 pounds. First attack of appendicitis about one year previous. Second attack November 3, 1908. A. M. Doctor called P. M.—gave hypodermic of rest medicine. Dr. W. T. Jones called next A. M., and I saw patient about 11 A. M. Pulse about 78. Temperature 98.8 F., no pain, no special rigidity, tongue slightly furred—we advised operation, but patient refused, said he would go to business next day he was feeling so well.

Feeling there was serious danger in this case, the attending physician, Dr. Jones called again in a few hours and found patient with pulse above 100, temperature nearly 104 F., but not suffering any pain and no rigidity of muscles.

I was called again at 5 P. M. We both urged immediate operation. After considerable persuasion patient consented and was given hyposcine 1-100 gr. morphine, 1-4 gr. hypodermically at once and moved to Tabernacle Infirmary in a chair, being only two squares away. The pain returned shortly after the hypodermic and became intense before anaesthetic was given, so patient begged for it, to get relief from the severe pain. Incision in right ingunial region, muscle splitting method, pus and flaky peritoneal fluid escaped as soon as abdomen was opened, omentum adherent but easily separated. Caecum bound down by old adhesions from previous attack, appendix retrocaecal—separating caecal adhesions found three gangrenous spots in it, size of thumb nail, appendix could not be brought up into incision, was gangrenous ligatures cut, so applied two long clamps to base of appendix, placed three large drainage tubes, two retrocaecal ones in right side of pelvis and wound dressed, provisional silk worm gut satures being introduced.

Hypodermoclisis used, patient placed to bed in Fowler's position and Murphy's proctoclysis used for 36 hours.

Next A. M. when nurse and intern were out of room patient got out of bed and walked across room to urinate to prevent use of catheter.

Forceps loosened in 36 hours, removed in 48 hours, fecal discharge present tissues sloughed from discharge, wound healthy, granulating and filling up in two weeks, but became infected in some way, temperature and pulse ran up, sloughing returned. With large dose quinine in rectum 10 to 20 c. c. antistreptococcic serum daily and supporting treatment, trouble soon subsided fecal fistula closed, no hernia, made good recovery, small scar, now regained usual weight. This patient was once operated upon by Sir Joseph Lister for injury to left leg, which is shortened and stiff and interfered with handling patient in this illness.

The thick abdominal walls and excessive amount of fat interfered with healing process.

In the last two cases the provisional silk worm gut sutures that were introduced at operation could not be utilized later on account of severity of infection, fecal discharge and sloughing, being compelled to have free drainage to save life of patients.

In the last few years Ochsner, Murphy and Fowler have given us life saving measures in handling these desperate cases. The knowledge of the absorptive powers and motion of the peritoneum being least in the pelvis, greatest at the diaphragm has demonstrated the great value of the Fowler's position. Ochsner's demonstration that food or fluids in stomach set up peristaltic action of bowels proves the necessity of not feeding or filling stomach in cases of appendicitis and at times may necessitate washing out stomach with stomach tube. Murphy's phenomenal success in the treatment of fulminating peritonitis has demonstrated the value of the relief of tension, free drainage and flushing the tissues by procto-clysis. A minimum amount of work. a maximum amount of drainage without flushing out of abdomen spreading infection, or mopping out to produce traumatism.

Every surgeon should familiarize himself with Murphy's method of proctoclysis in order to use it properly and effectively. He states that "next to the conservative technique of the operative procedure, proctoclysis is second in importance as a life-saver. It rapidly restores blood pressure, it improves capillary circulation, it quiets the thirst, it eliminates the septic products and increases the excretions. All the details are simple, but they must be carried out with precision to secure the best results."

Murphy also urges the necessity of keeping this class of patients in Fowler's position before and after operation and as much so during operation as possible and while being conveyed to the hospital.

The method of treating the stump of the appendix has resulted in death in some instances from hemorrhage so that some surgeons now advise ligating stump in all cases where possible to do so. I have been as I believe obviating that danger by an extra bite or turn of the purse-string sature, forming a loop surrounding base of meso-appendix including arterial blood supply to appendix, so that when suture is tied the blood supply will be constricted.

In pelvic surgery in the female we are often confronted with the necessity to decide whether to remove or not to remove the appendix.

This will always be a question of personal equation and the wish of the patient.

Where the appendix is diseased and condition of patient will admit there should be no question as to removal.

I always get consent of patient to remove appendix if diseased. I have in some cases refused to operate without such consent where I felt it was likely to be found diseased.

Where appendix is not diseased so far as we can tell and patient prefers not to have it removed or where the vital powers of patient have been severely taxed with other operative work and appendix not specially at fault, I leave it. There is one point in this connection that must not be entirely ignored and that is where we have a diseased appendix leucocytosis exists or nature develops local fortifications or barriers against infection and it is less danger then to remove the appendix than in normal conditions where no increased resistance has been developed.

This brings up the subject of leucocytosis with polynuclear increase or lencopnea as indications and contra-indications for operative work and as an index to the vitality or resisting powers of the patient in combatting infection. Much good work is being done along this line and gives promise of good results when the findings are coupled with the clinical history of the case. The future will demonstrate its limitations and value, with more definite rules for guidance if it is to be of practical value in every day work.

Case 1 in this report was one of tubercular degeneration of the appendix involving intestine and omentum by removal of a part of the diseased structures and disinfecting the remains, the recovery was complete and has remained so up to this time. The tubercular condition was probably contracted from husband who died previously of tuberculosis.

The third case was probably a commencing tubercular involvement of a previously irritated and diseased appendix, the condition of the caecum omentum, the lymphatic, extravisation of blood in operating, the general tenderness of abdomen, loss of flesh and living with sister who has pulmonary tuberculosis tend to confirm such a diagnosis.

Case two was one of early atrophy or want of development of appendix being only 24 years of age, it was not a senile change.

It was more interesting because of so many deaths of relatives due to appendicitis.

Case four illustrates a more common condition of retrocecal appendix without meso-appendix more difficult to remove, at times requiring transfixing base first then separating upward, concretions being common in such cases.

Case five illustrates the unusual condition of appendicitis due to thread worms, also its rapid dangerous development without previous indication or warning.

Cases six and seven illustrate what can be done in desperate cases by prompt action and utilizing the important principles of treatment advocated by Ochsner, Fowler and Murphy.

